# An Increase in Mean Platelet Volume/Platelet Count Ratio Is Associated with Vascular Access Failure in Hemodialysis Patients

**DOI:** 10.1371/journal.pone.0170357

**Published:** 2017-01-17

**Authors:** Dong Ho Shin, So Yon Rhee, Hee Jung Jeon, Ji-Young Park, Shin-Wook Kang, Jieun Oh

**Affiliations:** 1 Department of Internal Medicine, College of Medicine, Kangdong Sacred Heart Hospital, Hallym University, Seoul, Korea; 2 Department of Medicine, Graduate School of Medicine, Yonsei University, Seoul, Korea; 3 Department of Laboratory Medicine, College of Medicine, Kangdong Sacred Heart Hospital, Hallym University, Seoul, Korea; 4 Department of Internal Medicine, College of Medicine, Yonsei University, Seoul, Korea; 5 Hallym Kidney Research Institute, Hallym University, Seoul, Korea; Nagoya University, JAPAN

## Abstract

After stenosis of arteriovenous vascular access in hemodialysis patients, platelets play a crucial role in subsequent thrombus formation, leading to access failure. In a previous study, the mean platelet volume (MPV)/platelet count ratio, but not MPV alone, was shown to be an independent predictor of 4-year mortality after myocardial infarction. However, little is known about the potential influence of MPV/platelet count ratio on vascular access patency in hemodialysis patients. A total of 143 patients undergoing routine hemodialysis were recruited between January 2013 and February 2016. Vascular access failure (VAF) was defined as thrombosis or a decrease of greater than 50% of normal vessel diameter, requiring either surgical revision or percutaneous transluminal angioplasty. Cox proportional hazards model analysis ascertained that the change of MPV/platelet count ratio between baseline and 3 months [Δ(MPV/platelet count ratio)_3mo-baseline_] had prognostic value for VAF. Additionally, the changes of MPV/platelet count ratio over time were compared in patients with and without VAF by using linear mixed model analysis. Of the 143 patients, 38 (26.6%) were diagnosed with VAF. During a median follow-up of 26.9 months (interquartile range 13.0–36.0 months), Δ(MPV/platelet count ratio)_3mo-baseline_ significantly increased in patients with VAF compared to that in patients without VAF [11.6 (6.3–19.0) vs. 0.8 (-1.8–4.0), *P*< 0.001]. In multivariate analysis, Δ(MPV/platelet ratio count)_3mo-baseline_ was an independent predictor of VAF, after adjusting for age, sex, diabetes, hypertension, coronary artery disease, cerebrovascular disease, vascular access type, the presence of previous VAF, and antiplatelet drug use (hazard ratio, 1.15; 95% confidence interval, 1.10–1.21; *P*< 0.001). Moreover, a liner mixed model revealed that there was a significant increase of MPV/platelet count ratio over time in patients with VAF compared to those without VAF (*P*< 0.001). An increase in MPV/platelet count ratio over time was an independent risk factor for VAF. Therefore, continuous monitoring of the MPV/platelet count ratio may be useful to screen the risk of VAF in patients undergoing routine hemodialysis.

## Introduction

Vascular access failure (VAF) substantially contributes to morbidity and hospitalization in hemodialysis patients [1, 2]. Thrombosis is a leading cause of VAF and usually results from stenotic lesions in the venous outflow system [3, 4]. These lesions develop from progressive neointimal hyperplasia, whose pathogenesis is similar to that of a classic atheroma [5, 6]. Although the occurrences of VAF and atherosclerotic lesions may share some similar pathogenic mechanisms [7, 8], there have been few effective treatments to prevent VAF because of poor understanding of its pathogenesis [9]. Therefore, the initial step in managing hemodialysis patients is to identify patients with high risk for VAF and next step is to require aggressive prevention and intervention strategies.

Platelets play a central role in the formation of a classic atheroma, leading to peripheral arterial disease, coronary artery disease, and ischemic stroke [10–12]. Although circulating platelets are heterogeneous with respect to their size and reactivity [13], it is generally accepted that large platelets are metabolically and enzymatically more active, and have higher thrombotic potential than smaller ones [14, 15]. The mean platelet volume (MPV) is calculations performed by automated blood analyzers using either electrical impedance or optical fluorescence method. It reflects the average size of platelets in a blood sample [16, 17]. Therefore, considerable attention has been paid to MPV as possible marker of platelet function and activation. Various studies have demonstrated that elevated MPV was associated with a higher rate of restenosis after coronary angioplasty and a higher rate of major adverse cardiovascular events in patients with non-ST elevation acute coronary syndrome [18, 19].

In addition to the predictive value of the MPV, some studies have shown that platelet counts might also predict outcomes after myocardial infarction (MI) [20, 21]. Furthermore, in a study evaluating MPV and platelet count data, Ranjith et al. found that patients with acute coronary syndrome had lower counts and larger platelet volumes in comparison to those with stable angina [22]. Moreover, previous studies demonstrated an inverse relationship between MPV and platelet counts in a normal population [23–25]. These findings tended to focus on interpreting as a ratio rather than separate variable in MPV and platelet count. In a recent study, the MPV/platelet count ratio, but not MPV alone, was shown to be an independent predictor of 4-year mortality after MI [26]. However, little is known about the potential influence of MPV/platelet count ratio on vascular access patency in hemodialysis patients. Therefore, we investigated whether the increase in MPV/platelet count ratio over time has prognostic value for VAF in hemodialysis patients.

## Materials and Methods

### Ethics statement

This study was carried out in accordance with the Declaration of Helsinki and was approved by the Institutional Review Board (IRB) of the Kangdong Sacred Heart Hospital (Ref. 2015-11-007-001). Since the current study was retrospective and the subjects were de-identified, the IRB waived the need for written consent.

### Patients

The study was conducted at the dialysis clinic in Kangdong Sacred Heart Hospital, a 600-bed teaching hospital. Routine hemodialysis was performed in 178 patients between January 2013 and February 2016. Of these, 35 were excluded because they had bone marrow suppression due to chemotherapy for cancer (n = 3), they underwent hemodialysis for < 3 months at our dialysis clinic (n = 28), and they had cuffed central catheters (n = 4). Thus, 143 patients were included in this study. Those were already on hemodialysis at the time of study enrollment. Patients were censored at the time of hemodialysis therapy discontinuation for renal transplantation (n = 2), change to peritoneal dialysis (n = 2), death with a functioning access (n = 19), loss to follow-up (n = 2), or transfer to another dialysis clinic (n = 26).

### Definition

VAF was defined as thrombosis or a decrease of greater than 50% of normal vessel diameter, requiring either surgical revision or percutaneous transluminal angioplasty. The occurrence of VAF was confirmed from medical records. Cardiovascular disease was defined as a history of coronary, cerebrovascular, or peripheral vascular disease; coronary disease was defined as a history of angioplasty, coronary artery bypass grafting, myocardial infarction, or angina; cerebrovascular disease was defined as a previous history of transient ischemic attack, stroke, or carotid endarterectomy; and peripheral vascular disease was defined as a history of claudication, ischemic limb loss and/or ulceration, or peripheral revascularization procedure. During the follow-up period, patients who received antiplatelet agents such as aspirin, clopidogrel, or cilostazol were defined as those treated for > 3 months.

### Hemodialysis

All patients underwent routine hemodialysis 3 times a week using an SDS-20 dialysis machine (JMS Co., Ltd., Japan). Each hemodialysis session was performed for 3 to 4 hours using a dialyzer with a blood flow rate of 250 to 300 ml/min and dialysate flow of 500 ml/min.

### Data collection

Baseline characteristics, including demographic and clinical data such as age, gender, and comorbid conditions were obtained from medical records. In addition, laboratory parameters such as MPV level, platelet count, white blood cell (WBC) count, and hemoglobin (Hb) level were analyzed from blood samples using an autoanalyzer (ADVIA 2120 Hematology System, Siemens Healthcare Diagnostics, Forchheim, Germany). Routine chemistry was assessed by an autoanalyzer (Beckman Coulter AU5800, Beckman Coulter Inc., Brea CA, USA). Serum intact parathyroid hormone (PTH) concentration was evaluated using a commercially available two-sided immunoradiometric assay (ADVIA Centaur XP, Siemens Healthcare Diagnostics, Forchheim, Germany). Blood samples for measurement were collected pre-hemodialysis and most laboratory values, including MPV level and platelet count, were measured monthly for each patient. The time between drawing of blood samples and analysis of the specimens was less than 30 min. Kt/V was determined according to the procedure of Gotch [27].

### Statistical analyses

Statistical analyses were performed using SPSS 19.0 (SPSS Inc., Chicago, IL, USA) and R version 3.0.2 (http://cran.r-project.org/). Continuous variables were expressed as mean ±standard deviation (SD) and categorical variables as numbers (percentages). We evaluated the first event of VAF after study enrollment as a primary endpoint. Baseline characteristics are presented according to the occurrence of the primary outcome (VAF vs. non-VAF), and were compared for 2 groups using Student’s *t* test for continuous variables and the chi-square test for categorical variables. The MPV/platelet count ratio was expressed as fL/million platelets/cc blood. There was no event of VAF during the 3 months after the time of study enrollment. So, 3 months were chosen as the measurement point for correlation. MPV/platelet count ratio at 3 months minus MPV/platelet count ratio at baseline was considered the change in MPV/platelet count ratio at 3 months after study enrollment [Δ(MPV/platelet count ratio)_3mo-baseline_]. The prognostic value of Δ(MPV/platelet count ratio)_3mo-baseline_ for VAF was ascertained by Cox proportional hazards regression models that included all covariates with a *P*-value < 0.1 on univariate analysis. The predictive value for VAF was also analyzed by receiver operating characteristic (ROC) curve analysis with calculation of the area under the ROC curve (AUC). In addition, similar analyses were also performed at 4 months after study enrollment to confirm whether the correlation between VAF and MPV/platelet count ratio was maintained at other time point. The changes in MPV/platelet count ratio over time were compared between VAF and non-VAF groups using linear mixed model analysis. In this analysis, groups of VAF and non-VAF and follow-up time were treated as fixed effects, and each patient was treated as random effect such that each subject had a unique intercept and slope. All probabilities were 2-tailed and the level of significance was set at 0.05.

## Results

### Baseline characteristics according to the occurrence of VAF

The baseline characteristics of the study population are shown in Table 1. The mean age was 62.3 ± 11.4 years, and 62 (43.4%) were male. The median duration of dialysis was 32.7 months (interquartile range 16.4–74.2 months). Eight patients have total blood cholesterol levels of 240 mg/dL or higher. Of 143 patients, 38 patients developed VAF. However, there was no event of VAF during the 3 months after study enrollment. Of note, 55 patients had at least 1 VAF before study enrollment, and the frequency of VAF was 0.28 episodes per patient-year. Compared to patients without VAF, those with VAF were older (65.6 ± 10.6 vs. 61.1 ± 11.4 years, *P* = 0.04) and had a higher proportion of diabetes causing renal disease (97.4% vs. 53.3%, *P* < 0.001). Coronary artery disease (39.5% vs. 21.0%, *P* = 0.03), and arteriovenous graft (76.3% vs. 19.1%, *P* < 0.001) were more common in patients with VAF. In particular, the proportion taking antiplatelet agents was lower in patients with VAF (52.6% vs. 70.5%, *P* = 0.05). MPV/platelet count ratio at 3 months (58.5 ± 13.0 vs. 43.5 ± 10.6, *P* < 0.001) and Δ(MPV/platelet count ratio)_3mo-baseline_ [11.6 (6.3–19.0) vs. 0.8 (-1.8–4.0), *P* < 0.001] were significantly higher in patients with VAF. In addition, MPV/platelet count ratio at 4 months (57.7 ± 10.4 vs. 42.7 ± 7.8, *P* < 0.001) and Δ(MPV/platelet count ratio)_4mo-baseline_ [13.0 (6.3–18.9) vs. 0.2 (-5.6–4.0), *P* < 0.001] were also significantly higher in patients with VAF. In contrast, the systolic and diastolic blood pressure, serum Hb levels, platelet counts, MPV levels, cholesterol levels, albumin levels, calcium levels, phosphate levels, PTH levels, and Kt/V were comparable between the 2 groups.

**Table 1:** Baseline demographics, clinical characteristics, and biochemical variables according to the occurrence of VAF.

Variable	Total (n = 143)	Non-VAF (n = 105)	VAF (n = 38)	*P*-value
Demographic data				
Age (y)	62.3 ± 11.4	61.1 ± 11.4	65.6 ± 10.6	0.04
Male sex, n (%)	62 (43.4%)	51 (48.6)	11 (28.9)	0.06
Clinical data				
Duration of dialysis before study enrollment (months)	32.7 (16.4–74.2)	33.2 (17.6–81.5)	26.0 (15.9–66.8)	0.21
Primary renal disease				< 0.001
Diabetes, n (%)	93 (65.0)	56 (53.3)	37 (97.4)	
Non-diabetes, n (%)	50 (35.0)	49 (46.7)	1 (2.6)	
Comorbidity				
Coronary artery disease, n (%)	37 (25.9)	22 (21.0)	15 (39.5)	0.03
Cerebrovascular disease, n (%)	12 (8.4)	6 (5.7)	6 (15.8)	0.06
Peripheral artery disease, n (%)	1 (0.7)	1 (1.0)	0 (0)	0.99
Vascular access type				< 0.001
Arteriovenous graft, n (%)	49 (34.3)	20 (19.1)	29 (76.3)	
Arteriovenous fistula, n (%)	94 (65.7)	85 (80.9)	9 (23.7)	
The time from access construction to study enrollment (months)	35.7 (17.4–77.2)	36.2 (19.5–84.5)	27.0 (16.4–68.1)	0.12
The time from first use of the access to study enrollment (months)	32.7 (16.4–74.2)	33.0 (18.0–81.0)	26.0 (15.4–66.2)	0.21
Previous VAF	55 (38.5)	23 (21.9)	32 (84.2)	< 0.001
Systolic BP (mmHg)	149.7 ± 24.3	148.5 ± 24.2	152.9 ± 24.8	0.34
Diastolic BP (mmHg)	76.2 ± 11.3	76.5 ± 11.9	75.5 ± 9.5	0.66
MAP (mmHg)	100.7 ± 14.4	100.5 ± 14.9	101.3 ± 13.3	0.76
Antiplatelet drugs use, n (%)	94 (65.7)	74 (70.5)	20 (52.6)	0.05
Aspirin, n (%)	50 (35.0)	41 (39.0)	9 (23.7)	0.09
Clopidogrel, n (%)	35 (24.5)	26 (24.8)	9 (23.7)	0.90
Cilostazol, n (%)	3 (2.1)	2 (1.9)	1 (2.6)	0.79
Aspirin + clopidogrel, n (%)	6 (4.2)	5 (4.8)	1 (2.6)	0.58
Biochemical data				
Hemoglobin	9.8 (9.0–10.7)	9.7 (9.0–10.7)	9.9 (9.0–10.5)	0.77
Platelet (×10^3^/μL)	178.1 ± 40.6	179.9 ± 40.6	172.9 ± 40.8	0.37
MPV (fL)	7.4 ± 0.6	7.4 ± 0.6	7.3 ± 0.5	0.53
MPV/P ratio	43.8 ± 11.4	43.5 ± 11.3	44.9 ± 11.5	0.51
MPV/P ratio at 3 months	47.5 ±13.0	43.5 ±10.6	58.5 ± 13.0	< 0.001
Δ(MPV/P ratio)_3mo-baseline_	3.3 (-0.4–7.0)	0.8 (-1.8–4.0)	11.6 (6.3–19.0)	< 0.001
Albumin (g/dL)	3.7 ± 0.4	3.7 ± 0.4	3.6 ± 0.4	0.38
Triglyceride (mg/dL)	113.6 ± 82.5	109.5 ± 80.9	125.3 ± 86.9	0.33
Cholesterol(mg/dL)	132.0 (115.0–157.0)	131.0 (112.0–157.0)	134.0 (118.0–154.0)	0.83
Calcium (mg/dL)	8.5 ± 0.8	8.5 ± 0.8	8.4 ± 0.6	0.80
Phosphate (mg/dL)	4.8 ± 1.6	4.8 ± 1.7	4.7 ± 1.5	0.99
PTH (pg/mL)	234.2 (141.6–359.1)	246.4 (133.6–372.0)	200.8 (165.0–306.7)	0.73
Kt/V	1.5 ± 0.2	1.5 ± 0.3	1.5 ± 0.2	0.99

Note: values are expressed as median ± SD or median (interquartile range) or number (percentage).

Abbreviations: VAF, vascular access failure; BP, blood pressure; MAP, mean arterial pressure, MPV, mean platelet volume; PTH, parathyroid hormone.

Unit of MPV/P ratio is fL/million platelets/cc blood.

Δ(MPV/P ratio)_3mo-baseline_ was calculated as MPV/P ratio at 3months—MPV/P ratio at baseline.

### Correlation between changes in MPV/platelet count ratio and other parameters

Pearson’s correlation analysis revealed a significant positive correlation between Δ(MPV/platelet count ratio)_3mo-baseline_ and diabetes (r = 0.28, *P* < 0.001), arteriovenous graft (r = 0.35, *P* < 0.001), and cerebrovascular disease (r = 0.18, *P* = 0.03), respectively. In contrast, antiplatelet drugs use showed a significant inverse correlation with (ΔMPV/platelet count ratio)_3mo-baseline_ (r = -0.18, *P* = 0.04) (Table 2).

**Table 2:** Correlation between Δ(MPV/P ratio)_3mo-baseline_ and variables.

Variable	Δ(MPV/P ratio)_3mo-baseline_	
	*R*	*P*-value
Age (per 1 year)	0.05	0.52
Male	-0.09	0.29
Diabetes	0.28	< 0.001
Arteriovenous graft (vs. arteriovenous fistula)	0.35	< 0.001
Coronary artery disease	0.08	0.35
Cerebrovascular disease	0.18	0.03
Peripheral artery disease	-0.03	0.75
Antiplatelet drugs use	-0.18	0.04
Hemoglobin (g/dL)	-0.09	0.31
Albumin (g/dL)	-0.06	0.45
Triglyceride (mg/dL)	0.14	0.11
Total cholesterol (mg/dL)	0.02	0.79
Calcium (mg/dL)	-0.07	0.42
Phosphate (mg/dL)	-0.03	0.71
PTH (pg/mL)	0.02	0.82
Kt/V (per 1.0)	-0.01	0.87

Abbreviations: VAF, vascular access failure; MPV, mean platelet volume; PTH, parathyroid hormone.

Unit of MPV/P ratio is fL/million platelets/μL blood.

Δ(MPV/P ratio)_3mo-baseline_ was calculated as MPV/P ratio at 3months—MPV/P ratio at baseline.

### Increase in MPV/platelet count ratio and VAF

**Fig 1:**
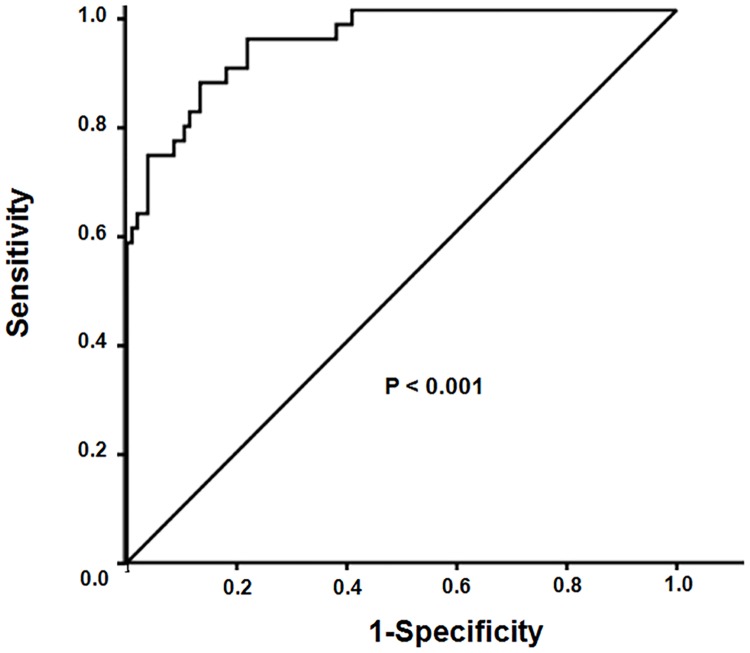
Receiver operating characteristic (ROC) curves of Δ(MPV/platelet count ratio)_3mo-baseline_ for VAF.

During the median follow-up of 26.9 months (interquartile range 13.0–36.0 months), 38 patients experienced VAF. The median time between study enrollment and the occurrence of VAF was 15.5 months (interquartile range 9.2–24.0 months). Univariate Cox regression analysis revealed that Δ(MPV/platelet count ratio)_3mo-baseline_, age, diabetes, cerebrovascular disease, arteriovenous graft, and previous VAF were significantly associated with VAF. In contrast, male sex correlated with a lower risk of VAF (Table 3). In multivariate analysis, Δ(MPV/platelet count ratio)_3mo-baseline_ remained a significant independent risk factor for VAF, even after adjusting for age, sex, diabetes, coronary artery disease, cerebrovascular disease, and vascular access type (hazard ratio [HR], 1.16; 95% confidence interval [CI], 1.11–1.22; *P* < 0.001 in Model 1). Further adjustment of Model 1 for the presence of previous VAF and antiplatelet drugs use (Model 2) did not attenuate the significant prognostic value of Δ(MPV/platelet count ratio)_3mo-baseline_ on VAF (HR, 1.15; 95% CI, 1.10–1.21; *P* < 0.001 in Model 2) (Table 4). In addition, diabetes, arteriovenous graft, and previous VAF were still significantly associated with VAF in the final multivariate model (Table 5). In addition, Δ(MPV/platelet count ratio)_4mo-baseline_ also remained a significant independent risk factor for VAF, even after adjusting for age, sex, diabetes, coronary artery disease, cerebrovascular disease, vascular access type, the presence of previous VAF, and antiplatelet drugs use (HR, 1.13; 95% confidence interval [CI], 1.07–1.19; *P* < 0.001) (S1 Table). ROC curves of Δ(MPV/platelet count ratio)_3mo-baseline_ for VAF are shown in Fig 1. AUC of Δ(MPV/platelet count ratio)_3mo-baseline_ was 0.94 (*P* < 0.001) (Fig 1). Of note, using ROC curves of Δ(MPV/platelet count ratio)_4mo-baseline_ for VAF, AUC of Δ(MPV/platelet count ratio)_4mo-baseline_ was 0.91 (*P* < 0.001).

**Table 3:** Cox proportional hazards analysis for VAF.

Variable	HR (95% CI)	*P*-value
Δ(MPV/P ratio)_3mo-baseline_ (per 1)	1.19 (1.15–1.24)	< 0.001
Age (per 1 y)	1.03 (1.00–1.07)	0.04
Male (vs. female)	0.49 (0.24–0.98)	0.04
Diabetes	25.29 (3.47–184.40)	0.001
Coronary artery disease	1.87 (0.98–3.58)	0.06
Cerebrovascular disease	2.37 (0.99–5.67)	0.05
Arteriovenous graft (vs. arteriovenous fistula)	8.57 (4.03–18.24)	< 0.001
Previous VAF	8.63 (3.61–20.64)	< 0.001
Antiplatelet drugs use	0.54(0.28–1.02)	0.06
Hemoglobin (g/dL)	0.94 (0.73–1.22)	0.65
Platelet (per 10^3^/ μL)	1.00 (0.99–1.01)	0.83
MPV (fL)	0.84 (0.49–1.45)	0.52
Albumin (g/dL)	0.54 (0.25–1.17)	0.12
Triglyceride (mg/dL)	1.00 (0.99–1.01)	0.23
Total cholesterol (mg/dL)	1.01 (0.99–1.01)	0.37
Calcium (per 1mg/dL)	0.42 (0.53–1.31)	0.42
Phosphate (per 1mg/dL)	1.00 (0.83–1.22)	0.98
PTH (per 1pg/mL)	0.99 (0.99–1.00)	0.92
Kt/V (per 1)	0.85 (0.24–2.99)	0.85

Abbreviations: VAF, vascular access failure; MPV, mean platelet volume; PTH, parathyroid hormone; HR, hazard ratio; CI, confidence interval.

Unit of MPV/P ratio is fL/million platelets/μL blood.

Δ(MPV/P ratio)_3mo-baseline_ was calculated as MPV/P ratio at 3months—MPV/P ratio at baseline.

**Table 4:** Multivariate Cox proportional hazards analysis for VAF.

Cox model	Δ(MPV/P ratio)_3mo-baseline_ (per 1)	
	HR (95% CI)	*P*-value
Unadjusted	1.19 (1.15–1.24)	< 0.001
Model 1	1.16 (1.11–1.22)	< 0.001
Model 2	1.15 (1.10–1.21)	< 0.001

Abbreviations: VAF, vascular access failure; MPV, mean platelet volume HR, hazard ratio; CI, confidence interval.

Unadjusted: crude relative risk

Model 1: adjusted for age, sex, diabetes, coronary artery disease, cerebrovascular disease, and vascular access type

Model 2: model 1 plus adjustment for previous VAF and antiplatelet drugs use.

Unit of MPV/P ratio is fL/million platelets/μL blood.

Δ(MPV/P ratio)_3mo-baseline_ was calculated as MPV/P ratio at 3months—MPV/P ratio at baseline.

**Table 5:** Multivariate Cox proportional hazards analysis for VAF (Model 2).

Variable	HR (95% CI)	*P*-value
Δ(MPV/P ratio)_3mo-baseline_ (per 1)	1.15 (1.10–1.21)	< 0.001
Age (per 1 y)	1.01(0.98–1.04)	0.64
Male (vs. female)	1.20 (0.53–2.69)	0.66
Diabetes	9.24 (1.15–74.14)	0.04
Coronary artery disease	1.74 (0.74–4.07)	0.20
Cerebrovascular disease	1.94 (0.69–5.45)	0.21
Arteriovenous graft (vs. arteriovenous fistula)	2.53 (1.02–6.26)	0.05
Previous VAF	3.14 (1.16–8.50)	0.02
Antiplatelet drugs use	1.29 (0.54–3.12)	0.57

Abbreviations: VAF, vascular access failure; MPV, mean platelet volume; HR, hazard ratio; CI, confidence interval.

Unit of MPV/P ratio is fL/million platelets/μL blood.

Δ(MPV/P ratio)_3mo-baseline_ was calculated as MPV/P ratio at 3 months—MPV/P ratio at baseline.

### Comparison of MPV/platelet count ratio over time in patients with VAF and without VAF

**Fig 2:**
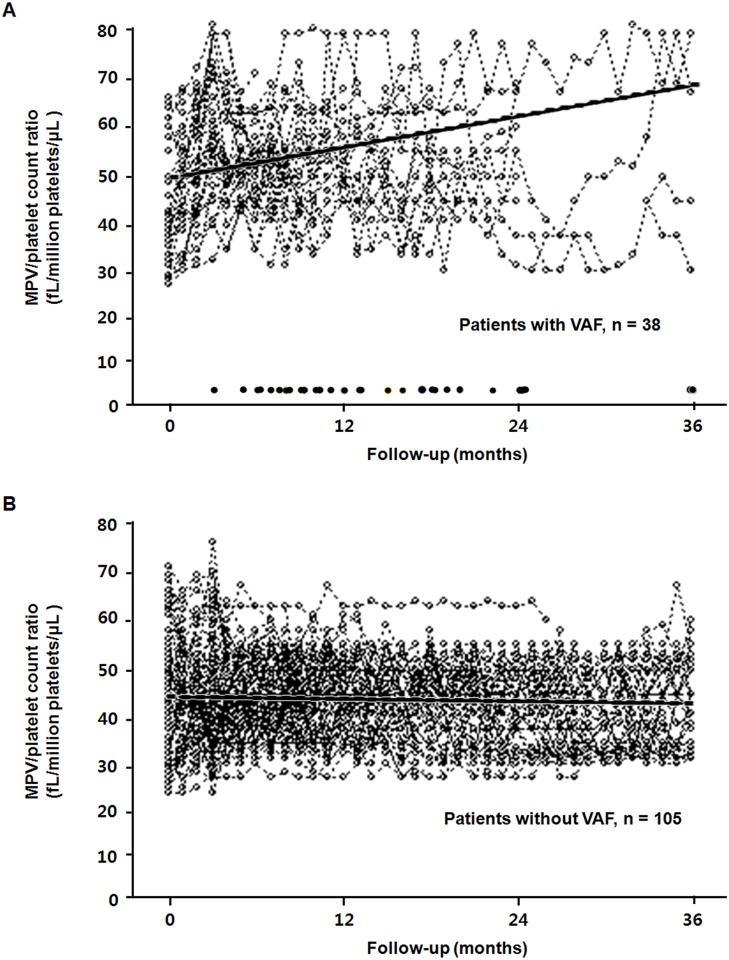
MPV/platelet count ratio over time in patients with VAF (A) and without VAF (B). Dash lines represent individual patient measurements, and darker solid lines represent predicted values. The linear mixed model revealed a significantly increased MPV/platelet count ratio over time in patients with VAF (A) compared with patients without VAF (B) (*P* < 0.001). A row of dots along the bottom of the plot (A) showed the time points at which vascular accesses failed.

The results of MPV/platelet count ratio during the study period are shown in Fig 2. Baseline MPV/platelet count ratio was comparable between patients with VAF and without VAF. However, the linear mixed model revealed a significantly increased MPV/platelet count ratio over time in patients with VAF compared with patients without VAF (*P* < 0.001) (Fig 2).

## Discussion

Several studies have shown that VAF is linked to many variables, including age, sex, diabetes, total cholesterol, cardiovascular disease, and vascular access type, along with many others [28–30]. This study showed that an increase in MPV/platelet count ratio over time was independently associated with VAF. To our knowledge, this is the first study to investigate that an increase in MPV/platelet count ratio over time was an independent risk factor for VAF in patients undergoing routine hemodialysis.

In our study, the prevalence of VAF was 0.28 episodes per patient-year, which was comparable to the value in previous reports [31, 32]. In addition, older age, diabetes, male sex, cardiovascular disease, and arteriovenous graft were associated with VAF in hemodialysis patients, which was consistent with other studies [28, 29, 33]. In particular, arteriovenous graft was an independent risk factor for VAF in multivariate analysis adjusted for such risk factors. This may be due to the accelerated intimal hyperplasia caused by flow turbulence, surgical trauma, compliance mismatch, and the release of growth factors from platelets that accumulate on the luminal surface at the graft-to-vein anastomosis [34–36]. Moreover, although there have been no large prospective studies that antiplatelet agents had significantly beneficial effect to prevent VAF, this study showed that use of antiplatelet agents was associated with a trend toward maintaining vascular access patency as demonstrated by other study [37]. Furthermore, several studies have recently suggested that dyslipidemia is a well-known risk factor of vascular disease in hemodialysis patients [38, 39]. However, in our study, serum cholesterol level was not associated with VAF. The low prevalence of patients with hypercholesterolemia (5.6%) in this study may explain the absence of a correlation between cholesterol level and VAF.

Although there are various monitoring and surveillance techniques to identify arteriovenous vascular access dysfunction, the choice is affected by many variables; chief among these are vascular access type, technology, effect of operator, and cost [40]. Therefore, it is imperative to develop simple, economical, and universal methods to screen for the risk of VAF in hemodialysis patients. One of things to deserve attention is to measure platelet reactivity since platelets play a major role in vascular disease. Interestingly, platelet dysfunction in uremic patients has been observed due to decreased platelet aggregation and impaired platelet adhesion. It may result from the disturbance of the platelet α-granules, changed Ca^+^ metabolism, decreased function of GP IIb/IIIa, and reduced binding of vWF and fibrinogen in uremic condition [41]. On the contrary, it has been demonstrated that in uremic patients, increased levels of phosphadidyserine, P-selectin, and fibrinogen receptor PAC-1 in platelets were associated with an increased risk of thrombosis [41]. Although little is known about the reasons why one uremic patient develops bleeding problem, while another tends to excessive thrombus formation, the general method is needed to measure platelet reactivity.

The MPV is a machine-calculated measurement of the average size of platelets found in blood and is typically included in blood tests as part of the CBC. In particular, it is increasingly recognized as an important marker of platelet activity [16, 17]. Large platelets contain more prothrombotic cytokines such as P-selectin, serotonin (5-hydroxytryptophan), ADP, and β-thromboglobulin in the form of α-granules. They also have more intracellular thromboxane A2 and express more adhesion receptors, glycoprotein Ib, and glycoprotein IIb-IIIa [42]. These factors contained in large platelets have a variety of effects in inflammation and endothelial function associated with vascular disease. In fact, various studies have shown the association between MPV and vascular disease. In a study by Choi el al., MPV independently predicted acetylcholine-induced coronary vasospasm [43]. In line with the finding, Berger et al. showed that increased MPV value was independently associated with peripheral artery disease, as defined by an ankle brachial index ≤ 0.90 in either leg [44]. In contrast, a large prospective study about association of MPV and the extent of coronary artery disease reported conflicting results. MPV was not related to platelet aggregation, the extent of coronary artery disease, or carotid intima-media thickness [45]. Thus, this parameter could not be considered a marker of platelet reactivity or a risk factor for coronary artery disease [45]. Interestingly, it could be explained by the finding that the increase in MPV might be a process driven by increased production of bone marrow-derived larger circulating reticulated platelets in the blood stream [46]. In fact, several studies showed that increased MPV correlated with both megakaryocyte ploidy and with the percentage of circulating reticulated platelets [47]. Therefore, these findings suggest that larger MPV may not indicate higher platelet reactivity but may be associated with reduced aggregation, since larger platelets may be not fully mature platelets. In contrast to this opinion, other studies demonstrated that larger circulating reticulated platelets were associated with prothrombotic potential [48, 49]. In addition, in a study by Cesari et al., the presence of a high rate of platelet turnover in acute coronary syndrome patients was an independent predictor of poor response to dual antiplatelet therapy [50], possibly due to the increased reactivity and to the presence of uninhibited cyclooxygenase (COX)-1 and COX-2 activity [51]. Although there were variety findings for the association between MPV and platelet function, another platelet volume index such as PDW was not at all related with the extend of coronary artery disease in a large study of patients undergoing coronary angiography [52]. In addition, different patient populations in the various studies of MPV might induce conflicting finding [53].

MPV and the platelet count are usually inversely related [23]. Therefore, an increased MPV/platelet count ratio reflects increased MPV and a low platelet count. Moreover, a decrease in platelet count may indicate activation of the coagulation system. In fact, several studies showed that a low platelet count come along with increased glycoprotein VI and inflammatory markers, which potentially reflects increased activation of platelets in cardiovascular disease [54, 55]. Interestingly, Williams et al. showed that heparin induced thrombocytopenia might contribute to recurrent ischemic events in patients with acute coronary syndrome [56]. Thus, the MPV/platelet count ratio could have greater diagnostic value than MPV alone in predicting platelet reactivity. In fact, Han et al. reported that the MPV/platelet count ratio could be a more useful predictor with greater sensitivity and specificity for detection of deep vein thrombosis than MPV alone [57].

Our study had several limitations. First, this type of study was a retrospective observational design, and the observational nature limits our ability to draw causal inference that an increase in MPV/platelet count ratio over time was an independent risk factor for VAF in patients undergoing routine hemodialysis. Second, the nature of single-center study caused the selection of patients was limited. However, because the surgical approach of vascular access creation and the care protocol of vascular access were similar in these patients, the impact of these factors on VAF could be minimized. Third, because ethylenediaminetetraacetic acid (EDTA) used as an anticoagulant, time-dependent swelling of platelets could be observed in samples. However, the analysis was performed within 30 min after vein puncture to minimize the effects on MPV.

In conclusion, this study showed that an increase in MPV/platelet count ratio over time was independently associated with VAF in hemodialysis patients. Although further studies are needed to elucidate the role of the MPV/platelet count ratio as a risk factor for VAF, the result suggests that continuous monitoring of the ratio may be useful to screen the risk for VAF in patients undergoing routine hemodialysis. In addition, a prospective trial testing the utility of antiplatelet agents is needed in the setting of high corrected MPV.

## Supporting Information

S1 Table Multivariate Cox proportional hazards analysis for VAF. (DOCX)
