# Supplementary material for: An Increase in Mean Platelet Volume/Platelet Count Ratio Is Associated with Vascular Access Failure in Hemodialysis Patients
Source: PLoS One. 2017 Jan 17;12(1):e0170357. doi: 10.1371/journal.pone.0170357 (PMC5240979; doi:10.1371/journal.pone.0170357)
Supplement: S1 Table — (DOCX) [file pone.0170357.s001.docx]

Supplement Table 1. Multivariate Cox proportional hazards analysis for VAF

| Variable | HR (95% CI) | *P*-value |
| --- | --- | --- |
| Δ(MPV/P ratio)_4mo-baseline_ (per 1) | 1.13 (1.07 – 1.19) | < 0.001 |
| Age (per 1 y) | 1.03 (0.99 – 1.07) | 0.14 |
| Male (vs. female) | 1.20 (0.54 – 2.66) | 0.66 |
| Diabetes | 8.60 (1.07 – 68.99) | 0.04 |
| Coronary artery disease | 1.46 (0.64 – 3.31) | 0.37 |
| Cerebrovascular disease | 1.65 (0.58 – 4.68) | 0.35 |
| Arteriovenous graft (vs. arteriovenous fistula) | 2.66 (1.10 – 6.45) | 0.03 |
| Previous VAF | 2.62 (1.01 – 6.93) | 0.05 |
| Antiplatelet drugs use | 0.97 (0.47 – 2.04) | 0.94 |

Abbreviations: VAF, vascular access failure; MPV, mean platelet volume; HR, hazard ratio; CI, confidence interval.

Unit of MPV/P ratio is fL/million platelets/μL blood.

Δ(MPV/P ratio)_4mo-baseline_ was calculated as MPV/P ratio at 4 months - MPV/P ratio at baseline.
